# Seroprevalence of *Bartonella *spp. infection in HIV patients in Catalonia, Spain

**DOI:** 10.1186/1471-2334-8-58

**Published:** 2008-05-01

**Authors:** Immaculada Pons, Isabel Sanfeliu, María Mercedes Nogueras, Montserrat Sala, Manuel Cervantes, M José Amengual, Ferran Segura

**Affiliations:** 1 Infectious Diseases Program, Hospital de Sabadell Institut Universitari Parc Taulí UAB, Sabadell, Spain; 2 Medicine Department, Universitat Autonoma de Barcelona, Barcelona, Spain; 3 UDIAT Diagnostic Center Laboratory, Sabadell, Spain

## Abstract

**Background:**

Although the first clinical descriptions of *Bartonella *infection were associated with immunocompromised patient with bacillary angiomatosis, we currently know that this organism is directly involved in diseases affecting a large number of patients, regardless of their immune status. Cat scratch disease, hepatic peliosis, and some cases of bacteraemia and endocarditis, are directly caused by some species of the genus *Bartonella*. The purpose of this study was to determinate the prevalence of IgG antibodies against *Bartonella henselae *and *B. quintana *in HIV patients and to identify the epidemiological factors involved.

**Methods:**

Serum samples were collected from HIV patients treated at Hospital de Sabadell. Antibodies to *B. henselae *and *B. quintana *from 340 patients were examined by indirect immunofluorescence assay (IFA). Significance levels for univariate statistical test were determined by the Mann-Whitney U test and χ^2 ^test.

**Results:**

Of 340 patients, 82 were women and 258 men, with a median age of 42.21 ± 10.35 years (range 16–86 years). Seventy-six (22.3%) patients reacted with one or more *Bartonella *antigens. Of all the factors concerning the seroprevalence rate being studied (age, sex, intravenous drugs use, alcohol consumption, CD4 levels, AIDS, HCV, HBV, residential area), only age was statistically significant.

**Conclusion:**

A high percentage of HIV patients presents antibodies to *Bartonella *and is increasing with age.

## Background

The spectrum of *Bartonella *infections has expanded rapidly since the first HIV- infected patient with unusual, vascular proliferative lesions of bacillary angiomatosis (BA) was described in 1983 [1]. Of the 19 species of *Bartonella *described until now, only 10 were acknowledged as human pathogen species; *B. bacilliformis*, *B. quintana*, and *B. henselae*, are the most frequently described species [2-4], while *B. elizabethae, B. vinsonii, B. washoensis, B. grahamii, B. clarridgeiae, B. koehlerae and B. alsatica *were recently identified as responsible for a few cases of human infections [5-10]. Cat scratch disease (CSD), hepatic peliosis and some cases of bacteraemia and endocarditis are directly caused by some species of the genus *Bartonella *[11-14]. To determine the real incidence of *Bartonella *infections, we must study the seroprevalence in the general population and the principal reservoirs and vectors of infection transmission. The results yielded by different studies on seroprevalence vary depending on the type of population under study; thus, the research conducted on collective groups that present special characteristics or associated risk factors present a higher prevalence than that found in studies carried out on the normal population.

In patients with addiction to parenteral drugs, it ranged from 15% to 47.5% [15,16]; in patients with HIV infection, it varied between 17.3% and 40% [17,18]. The objective of this study was to evaluate the prevalence of *Bartonella *infections in patients with HIV in our catchment area and to assess related factors.

## Methods

### Geographical area

The study was undertaken in Vallès Occidental (Catalonia), a predominantly urban area near the coast in the northeast of Spain.

### Samples

The collection of samples took place over a 10-month period, from October 2004 to July 2005. The sample included adults and children treated at Hospital de Sabadell (Vallès Occidental, an area near Barcelona, Spain), where most of the patients diagnosed with HIV from the region are treated (cathment area 407.763 inhabitants). They were attended in periodical CD4 follow-up. Serum samples were collected from these patients at their scheduled follow up visits. The residential area was determined considering the number of inhabitants who lived in the municipalities. In fact, municipalities with < 50.000 inhabitants were considered semirural areas, and >50.000 were regarded as urban areas. Demographic information was obtained from computerized clinic record. Information about alcoholism, drug use, CD4 levels, HCV and HBV serology, was available from computerized records for patients who regularly received care at the clinic and was obtained by chart review for some patients. Informed consent was obtained from adult participants and from the parents of minors.

### Serological technique

The sample of heparinized blood was sedimented (centrifugation of 5 ml samples of blood at 1.500 rpm for 10 minutes), and the supernatant was collected and stored at -80°C until used. Human serum samples were evaluated by indirect immunofluorescence assay (IFA). We used commercial slides (*Bartonella *IFA IgG. Focus Technologies, Inc., Herndon, VA) to determine antibodies to *Bartonella *spp. The kit for detecting IgG antibodies that employees Vero cells infected with either *B. henselae *or *B. quintana *was used according to the manufacturer's instructions. The serum samples were initially diluted 1/64. Any serum samples found to be positive at the initial dilution were further titrated. Positive and negative controls were included in each test. We considered specimens showing no fluorescence at IgG titers of 1/64 as negatives and specimens with bright fluorescence in a dilution of 1/64 or greater as positive. The intensity of each specific fluorescent test was subjectively evaluated and independently graded by two of the authors [19].

### Data analysis

Statistical analysis was performed using statistical package SPSS for Windows, Release 13.0.1 (standard version; SPSS, Inc., Chicago, IL). Significance levels for univariate statistical test were determined by the Mann-Whitney U test, χ^2 ^test and Fisher exact test. A *p *value of 0.05 or less was considered to be significant in all statistical tests used.

## Results

Of the 340 subjects, 82 (24.1%) were women and 258 (75.9%) men. Mean age was 42.21 ± 10.35 years (range 16–86 years). One hundred and ninety four (57%) and 146 (43%) patients lived in urban and semirural- rural areas, respectively. One hundred and ninety-six (57.6%) patients consumed or had consumed intravenous drugs, whilst 97 (28.5%) presented an excessive intake of alcohol. 13.8% of patients presented CD4 levels below or equal to 200 cell/ml at the time of the study. Of all the patients being followed-up, 102 (30%) had been diagnosed with AIDS. 50% of the patients were infected with the hepatitis C virus, whereas only 5% was infected with the hepatitis B virus (Table 1). None of the patients enrolled in this study presented neither a past history of diseases caused by exposure to *Bartonella* spp nor bartonellosis symptoms. Antibodies to *Bartonella* species were highly prevalent in this group of HIV patients; 76 (22.3%) of the samples reacted with at least 1 *Bartonella* antigen, 32 (42.1%) of the positive samples reacted with only *B. henselae* antigen, one sample (1.3%) reacted with only *B. quintana*, and 43 (56.6%) with both. In 34 serum samples, the titers obtained did not allow for differentiation between the 2 *Bartonella* species, as the specimens presented the same titer or one dilution as difference only. In the 9 remaining serum samples, the titers obtained for *B. henselae* were clearly superior (2 or more dilutions as difference) (Table 2). Fifty-seven patients (75%) were positive at a titer of 1/64, 10 (13.1%) at 1/128, 5 (6.6%) at 1/256, 2 patients (2.6%) at 1/512 and 2 patients (2.6%) were positive at a titer of 1/1024. The antibody titer was specific for *B. quintana* in one case only, with a titer of 1/128.

**Table 1:** HIV patients description and serologic results

**Characteristic**	**N total (%) (340 patients)**	**Positive to *Bartonella *N (%) (76 patients)**	**Negative to *Bartonella *N (%) (264 patients)**	**P-value**
**Sex**				0.611
Female	82 (24.1)	20 (26.3)	62 (24.4)	
Male	258 (75.9)	56 (73.7)	202 (78.3)	
**Immunitary state**				0.368
≤ 200 cell/ml	47 (13.8)	7 (9.2)	40 (15.2)	
> 200 cell/ml	293 (86.2)	69 (90.8)	224 (84.8)	
**AIDS ^1^**				0.820
Yes	102 (30)	22 (28.9)	80 (30.3)	
No	238 (70)	54 (71.1)	189 (69.7)	
**Drugs abuse**				0.401
Yes	196 (57.6)	47 (61.8)	149 (56.4)	
No	144 (42.4)	29 (38.2)	115 (43.6)	
**Alcohol**				0.704
Yes	97 (28.5)	23 (30.2)	74 (28.0)	
No	243 (71.5)	53 (69.7)	190 (72)	
**HBV ^2,3^**	17 (5)	3 (3.94)	14 (5.3)	0.449
**HCV^4^**	170 (50)	39 (51.3)	37 (48.7)	0.795
**Municipality (inhab)**				0.667
>50000	194 (57.0)	45 (59.2)	149 (56.4)	
<50000	146 (43.0)	31 (40.8)	115 (43.6)	
**Transmission group**				0.390
Heterosexual	62 (18.2)	11 (14.5)	51 (19.3)	
Homosexual	15 (4.4)	1 (1.3)	14 (5.3)	
IDU ^5^	194 (57.1)	47 (61.8)	147 (55.7)	
Bisexual	5 (1.5)	2 (2.6)	3 (1.1)	
Vertical	3 (0.9)	0 (0)	3 (0.9)	
Unknown	61 (18)	15 (19.7)	46 (17.4)	

1: AIDS criteria: Centers for Disease Control and Prevention (CDC) category C; 2: Hepatitis B virus infection; 3: Fisher Exact test; 4: Hepatitis C virus infection ; 5: Injection drug user.

**Table 2:** Pattern of IgG antibody titers to *B. henselae *and *B. quintana *in the study population (N= 340)

*B. quintana *titers				
*B. henselae titers*	<1/64	1/64	1/128	1/256
<1/64	264	0	1	0
1/64	28	29	0	0
1/128	4	6	0	0
1/256	1	5	0	0
1/512	0	1	1	0
1/1024	0	0	1	1

24.4% of women and 21.7% of men presented antibodies to *Bartonella* spp. A statistically significant increase of seropositivity against *Bartonella* spp. was observed as patient age increased (p < 0.05). Twenty-three (30.2%) patients with positive serology for *Bartonella* presented a past history of alcohol abuse. Of the 76 patients with positive serology, 47 (61.8%) were addicted to parenteral drugs and 22 (28.9%) had, at some time, being diagnosed with AIDS. Thirty-nine patients presented co-infection between *Bartonella* and HCV, whereas in 3 patients it was between *Bartonella* and HBV. No differences were found regarding the way of transmission of the human immunodeficiency syndrome.

## Discussion

Preliminary estimates of the prevalence of antibody to *Bartonella *among apparently healthy humans range from 5.88% to 24.7% [17,20]. In a study carried out on a healthy population sample from the same area (83 were men and 78 women, and the mean age with positive serology was 45.18 ± 14.26 years), the seroprevalence was 10.6% [21], a figure that is very similar to that reported in other studies. The incidence of *Bartonella*-associated disease among HIV- infected people is less known. The studies carried out show seroprevalence rates that range from 16 to 40% [18,22].

Epidemiological studies identified the major risk factors for acquiring *Bartonella *infections. *B. quintana *infection is associated with exposure to the human body louse, *Pediculus humanus *[13,23], and homeless conditions [23,24], whereas the major risk factor associated with *B. henselae *infection is cat exposure, especially cat scratches and fleas [13,25-27]. *B. henselae *seroprevalence in cats in our area was 29.6%, this result confirmed the presence of infection in the main reservoir of this microorganism [28].

*B. henselae *infection, as in other infections caused by fastidious bacteria, gives rise for concern given the complexity of the diagnosis and the variety of presenting clinical pictures, mainly in HIV-infected patients [29]. In fact, coetaneous BA lesions can be clinically indistinguishable from Kaposi's sarcoma, being differentiated by biopsy only. Hepatic disease caused by *Bartonella *can be indistinguishable from others infectious or malignant conditions that cause hypodense lesions demonstrable by abdominal CT [30]. All these factors make serology as a useful tool for the diagnosis of acute infections caused by *Bartonella *spp. However, it must be taken into account that there can be important limitations when dealing with HIV infected patients [31,32]. The serologic response in patients HIV with a good immunological status could be similar to that of the normal population, whereas possibly in situations of advanced immunodepression or in bacillary angiomatosis, the serologic response could show as a false negative [33].

Our study highlights in the first place an antibody seroprevalence of 22.4% to *Bartonella *species in HIV patients, which is superior to that observed in the healthy population [13,19,25], with the exception of that described in some other studies [25]. Some authors consider HIV patients as a risk group for *Bartonella *infection [34,35] (characteristic personal and hygienic habits); however, other authors do not consider this infection risk to be greater [20,34].

In our experience, a previous study carried out on a healthy population sample from the same area show a seropositive rate of 8.6% [21]. This study included 55 children and 161 adults (≥ 18 years). If children were not considered, the seroprevalence was 10.6%. In view of our HIV-infected patients, predominantly adult, seems to be higher, it could be considered HIV-infection as a risk factor.

Taking into account other studies, this difference among populations might be due to the fact that in HIV patients the infection can last longer or that these patients might become in contact more easily with some of the factors that have a direct impact on increased *Bartonella *infection, such as contact with cats (main reservoir of *B. henselae*) [16,17,24] or that they spend most of their time on the street [24,34]. However, these informations are not available in our study. We could attribute the high rate of seropositivity observed to false positives that are not directly due to HIV infection (to date no crossed- reactions have been observed between both); however, there are studies describing crossed reactions with *Coxiella burnetii *[32,35]. On the other hand, other studies suggest that HIV-infected patients present a higher risk of Q fever than immunocompetent patients [36]. The different factors that might play an important role in the transmission of *Bartonella *spp must also be taken into account. 57.6% of seropositive patients were intravenous drug users, these findings are most likely a result of several conditions that are frequent among IVDUs: living conditions with a high probability of close contact with bacteriaemic cats, fleas, lice, and other potential vectors, repeated parenteral exposures, insufficient medical care, and malnutrition [19,33,34,37]. Other studies do not conclude that drug use might play such an important role [20].

An excessive consumption of alcohol constitutes an elevated risk factor for infection, not only for its capacity to physiologically deteriorate regular alcohol abusers (liver alterations, neurological disorders, etc.), but because in the majority of cases drinking can affect people at low socioeconomic levels (homeless) who might be in closer contact with infection-transmitting vectors [38,39].

Of special interest in these patients was to find out whether the risk for infection increased with immunodepression. In our experience, we were unable to detect a greater incidence among patients who had been diagnosed with AIDS at some given time. In like manner, we were also unable to detect a greater infection incidence in patients with levels < 200 lymphocytes CD4/μl, in contrast with what has been reported by some authors who claim that the prevalence of antibodies against *B. henselae *is inversely proportional to the number of CD4 lymphocytes [30,35].

Our study presented only a 13.8% of patients with low CD4 levels, which could significantly alter our results. If we take into account that HIV-induced immunodepression does not appear to significantly modify the serological response to *Bartonella *spp., serology could be useful in the diagnosis of diseases associated with these microorganisms in HIV-infected patients (acute infection and population-based epidemiological studies). PCR techniques might be of help to diagnose *Bartonella *infection in patients who are more immunodepressed, especially when the infection is subclinical. *Bartonella *infections are substantially underrecognized because of their non-specific symptomatology.

## Conclusion

In conclusion, the seroprevalence of *Bartonella *spp. among HIV infected patients is greater than that of the healthy population of the same area and thus *Bartonella *infection should be considered in HIV patients.

## Competing interests

Immaculada Pons, Isabel Sanfeliu, María Mercedes Nogueras, Montserrat Sala, Manuel Cervantes, M José Amengual and Ferran Segura : The authors declare that they have no competing interests.

## Authors' contributions

IP carried out the analysis and interpretation of data, serological technique and the preparation and revision of manuscript.

IS participated in the study concept and design, serological technique and revision of manuscript.

MMN participated in the analysis of data and revision of manuscript.

MS participated in acquisition of epidemiological and clinical data.

MC participated in acquisition of epidemiological and clinical data.

MJA participated in the acquisition of data from laboratory.

FS participated in the study concept and design, and revision of manuscript.

## Pre-publication history

The pre-publication history for this paper can be accessed here:


